# Recently Investigated Natural Gums and Mucilages as Pharmaceutical Excipients: An Overview

**DOI:** 10.1155/2014/204849

**Published:** 2014-04-07

**Authors:** Pritam Dinesh Choudhary, Harshal Ashok Pawar

**Affiliations:** Dr. L.H. Hiranandani College of Pharmacy, Smt. CHM Campus, Opp. Ulhasnagar Railway Station, Ulhasnagar, Maharashtra 421003, India

## Abstract

Due to advances in drug delivery technology, currently, excipients are included in novel dosage forms to fulfil specific functions and in some cases they directly or indirectly influence the extent and/or rate of drug release and drug absorption. Recent trends towards use of plant based and natural products demand the replacement of synthetic additives with natural ones. Today, the whole world is increasingly interested in natural drugs and excipients. These natural materials have many advantages over synthetic ones as they are chemically inert, nontoxic, less expensive, biodegradable, and widely available. This review discusses majority of the plant-derived polymeric compounds (gums and mucilage's), their sources, chemical constituents, uses, and some recent investigations as excipients in novel drug delivery systems.

## 1. Introduction

In recent years, polymers those are derived from plant origin have evoked tremendous interest because of their diverse pharmaceutical applications such as diluent, binder, disintegrant in tablets, thickeners in oral liquids, protective colloids in suspensions, gelling agents in gels, and bases in suppository [1]. They are also used in cosmetics, paints, textiles, and paper making [2]. These natural gums and mucilages are preferred over the synthetic ones because they are biocompatible, cheap, and easily available than the synthetic ones. Also the natural excipients are preferred on the synthetic and semisynthetic ones because of their lack of toxicity, low cost, soothing action, availability, and nonirritant nature of the excipients [3–6]. Demand for these substances is increasing and new sources are being developed. India, because of its geographical and environmental position, has traditionally been a good source for such products among the Asian countries.

### 1.1. Gums and Mucilage's

Gums are considered to be pathological products, formed by giving injury to the plant or due to unfavourable conditions, such as drought, by breakdown of cell walls (extra cellular formation: gummosis). Mucilages are generally normal products of metabolism (physiological products), formed within the cell (intracellular formation). Gums readily dissolve in water, whereas, mucilage form slimy masses. Both gums and mucilages are plant hydrocolloids yielding mixture of sugars and uronic acids on hydrolysis [7].

Classification is based on source:

- marine origin/algal (seaweed) gums: agar, carrageenans, alginic acid, and laminarin;
- plant origin:
  - shrubs/tree exudates: gum arabic, gum ghatti, gum karaya, gum tragacanth, and khaya and albizia gums;
  - seed gums: guar gum, locust bean gum, starch, amylose, and cellulose;
  - extracts: pectin, larch gum;
  - tuber and roots: potato starch;
- animal origin: chitin and chitosan, chondroitin sulfate, and hyaluronic acid;
- microbial origin (bacterial and fungal): xanthan, dextran, curdian, pullulan, zanflo, emulsan, Baker's yeast glycan, schizophyllan, lentinan, krestin, and scleroglucan.

### 1.2. Isolation and Purification of Gums and Mucilage's

Mucilage can be extracted from plant parts by various methods like heating, solvent precipitation, and microwave assisted extraction. The easiest method is solvent precipitation. In this method the part of the plant containing gum/mucilage is selected followed by drying, grinding, and sieving of that plant part. This is then stirred in distilled water and heated for complete dispersion in distilled water and kept for 6–8 h at room temperature. The supernatant is obtained by centrifugation. The residue is then washed with water and the washings are added to the separated supernatant. Solvent for precipitation is selected and, finally, the supernatant is mixed with twice the volume of precipitating solvent by continuous stirring. The precipitated material is washed with distilled water and dried at 50–60°C under vacuum. Plant material must be treated with petroleum ether and chloroform (to remove pigments and chlorophyll) and then with distilled water [8, 9].

### 1.3. Characterization of Gums and Mucilage's

Preliminary confirmatory tests for dried gums and mucilage powders are summarised in Table 1 [10].

For characterization, analytical techniques can be classified according to the type of information generated.

*Structural.* Gums and mucilages are polysaccharides and they contain sugars. So, confirmation of different sugars present can be done by chromatography (TLC/HPLC) and structure elucidation can be carried out by FTIR, mass, and NMR spectroscopy.

*Purity.* To determine the purity of the selected gum and mucilage, tests for alkaloids, glycosides, steroids, carbohydrates, flavonoids, terpenes, amino acids, saponins, oils and fats, and tannins and phenols are carried out. 

*Impurity Profile.* Suitable analytical techniques can be used for testing of impurities.

*Physicochemical Properties.* Colour, odour, taste, shape, texture, touch, solubility, pH, swelling index, loss on drying, hygroscopic nature, angle of repose, bulk and true densities, porosity, and surface tension can be estimated. The microbial load and presence of specific pathogens are also determined. Gums and mucilages are highly viscous in nature. So, the rheological properties of excipients are important criteria for deciding their commercial use.

*Toxicity.* The acute toxicity of gums and mucilages are determined by fixed-dose method as per OECD guideline no. 425. [11].

## 2. Some Recently Investigated Natural Gums and Mucilages

### 2.1. Abelmoschus Gum

The okra gum is obtained from the fresh fruits of the plant* Abelmoschus esculentus* (family* Malvaceae*). The okra polysaccharide contains the major polysaccharide componentdiffering widely in the molar ratios of galactose, galacturonic acid, and rhamnose and with some fractions of glucose, mannose, arabinose, and xylose [12]. Mucilage from the pods of* Abelmoschus esculentus* is evaluated for its safety and suitability as suspending agent. Mucilage extracted was found to be nontoxic and was used for formulation of paracetamol suspension. The mucilage was found to be superior suspending agent than tragacanth and its suspending efficiency was similar to sodium CMC [13]. Mucilage was also evaluated for its disintegrating property. Various concentrations of the mucilage were used and batches of tablets were formulated and evaluated for dissolution, wetting time, and disintegration time. The study revealed that* Abelmoschus esculentus *mucilage powder was effective as disintegrant in low concentrations (4%) [14]. Gum of* Abelmoschus esculentus* is used as a polymer for the development of a gastric floating dosage form. In this study tablet batches were prepared using* Abelmoschus esculentus *mucilage and HPMC E15 in different combinations. It was seen that formulation containing* Abelmoschus esculentus *mucilage had poor floating capacity but showed sustained release, whereas formulation containing HPMC had better floating capacity but showed poor sustained release of the drug, so in all it was seen that formulation containing okra mucilage with HPMC gave better floating property as well as better sustained release of the drug [15]. Okra polysaccharide as a microbiallytriggered material for colon targeted tablet formulation and also as the carrier. The observations drive to conclude that the okra polysaccharide under investigation has the potential to carry the drug almost intact to the intended site, that is, colon where it undergoes degradation due to the presence of anaerobic microbes [16].

### 2.2. Albizia Gum

Albizia gum is obtained from the incised trunk of the tree* Albizia zygia* (Family* Leguminosae*). It consists of *β*-1-3-linked D galactose units with some ß1-6-linked D-galactose units. Albizia gum has been investigated as a possible substitute for gum arabic as a natural emulsifier for food and pharmaceuticals [17, 18]. These gums were tried as coating materials in compression-coated tablets, which degraded, by the colonic microflora, thereby releasing the drug [19].

### 2.3. Tamarind Seed Polysaccharide

Tamarind xyloglucan is obtained from the endosperm of the seed of the tamarind tree,* Tamarindus indica* (family* Fabaceae*). Tamarind gum is a polysaccharide composed of glucosyl : xylosyl : galactosyl in the ratio of 3 : 2 : 1. The polysaccharide obtained from tamarind seeds was made use of in formulating matrix tablets by wet granulation technique and was evaluated for its drug release characteristics. Tablets were prepared using different concentration of the polymer. Increase in polymer content decreased drug release [20]. Potentials of tamarind seed polysaccharide as a biodegradable carrier for colon specific drug delivery was studied. It was seen that the matrix tablets prepared by using tamarind gum were able to carry most of the drug to the colon and restrict the release in upper GIT [21].

### 2.4. Locust Bean Gum

Locust bean gum (LBG) (also known as carob gum) is obtained from the refined endosperm of seeds from the carob tree* Ceratonia siliqua* (family:* Leguminosae*). The polymer is neutral, slightly soluble in cold water and requires heat to achieve full hydration, solubilisation, and maximum viscosity [22]. The gum contains D-galacto-Dmannoglycan, pentane, proteins, and cellulose. Superdisintegrant property of this gum was studied by oral dispersible tablets containing locust bean gum and evaluating it against standard superdisintegrant that is croscarmellose sodium [23]. This gum has also been investigated for its controlled delivery property [24] and also as a compression coat which when applied over core tablets acts as a suitable carrier for colonic drug delivery, as it proves capable of protecting the core tablet and thus is a potential carrier for drug targeting to the colon [25].

### 2.5. Fenugreek Mucilage

Mucilageis obtained fromseeds of* Trigonella foenum-graceum *(family:* Leguminosae*).Its seeds contain a high percentage of mucilage and do not dissolve in water but form viscous tacky mass and swell up when exposed to fluids [26]. Gum contains mannose, galactose, and xylose. The mucilage obtained from fenugreek was found to be better release retardant compared to hypromellose at equivalent content [27].

### 2.6. Hibiscus Mucilage

Mucilage is obtained from fresh leaves of* Hibiscus rosa-sinensis *(family:* Malvaceae*). Mucilage of* Hibiscus rosa-sinensis *contains L-rhamnose, D-galactose, D-galacturonic acid, and D-glucuronic acid [28]. The use of its mucilage for the development of sustained release tablet has been reported [29].

### 2.7. Honey Locust Gum

The gum is obtained from the seeds of the plant* Gleditsia triacanthos* (family:* Leguminosae*). Seeds contain proteins, fats, carbohydrates, and fibres. Honey locust gum has been used to produce matrix tablets at different concentrations (5% and 10%) by wet granulation method [30].

### 2.8. Tara Gum

Tara gum is obtained from the endosperm of seed of* Caesalpinia spinosa* (family:* Leguminosae *or* Fabaceae*). The gum mainly contains galactomannans. The ratio of mannose to galactose in tara gum is 3 : 1 and produces highly viscous solutions, even at 1% concentration [31]. The use of tara gum as a controlled release carrier in the formulation of gastroretentive controlled release tablets due to swelling of the gum. Using tara gum in combination increases floating time of the dosage form thus showing good gastroretentive property [32]. Tara gum was also used formulation of emulsions [33].

### 2.9. Almond Gum

Almond gum is obtained from the tree* Prunus amygdalus* (family:* Rosaceae*). It is a water soluble gum extrudes from wounds on almond tree. Gum contains aldobionic acid, L-arabinose, L-galactose, D-mannose, etc. Almond gum contains different components which have emulsifying, thickening, suspending, adhesive, glazing, and stabilizing properties. Gum obtained from almond tree was studied for its binding property in tablet formulations. The drug release increased with almond gum when compared to synthetic gum concentration and the release mechanism was found to be non-Fickian diffusion. The almond gum was found to be useful for the preparation of uncoated tablet dosage form [34].

### 2.10. Cashew Gum

Cashew gum is the exudate from the stem bark of* Anacardium occidentale *(family:* Anacardiaceae*). The gum contains galactose, arabinose, rhamnose, glucose, glucuronic acid, and other sugar residues, while hydrolysis of the gum yields L-arabinose, L-rhamnose, D-galactose, and glucuronic acid [35]. Studies were performed on cashew gum for its gelling property. The gels prepared with 5.0% of mucilage were found to be ideal and comparable with a commercial preparation. The prepared gels did not produce any dermatological reactions. The gels were found to be stable with respect to viscosity, drug content, and physical appearance at all temperature conditions for 3 months [36]. Cashew gum was also studied for its binding property. In this study binding property of cashew gum was compared with acacia. It was observed that the disintegration time of the tablet increased with increase in concentration of cashew gum [37] and controlled release property wherein study showed that increase in the polymer ratio retarded the drug release to a greater extent [38].

### 2.11. Neem Gum

Neem gum is obtained from the trees of* Azadirachta indica *(family:* Meliaceae*). Gum contains mannose, glucosamine, arabinose, galactose, fucose, xylose, and glucose [39]. Studies were performed on neem gum for its binding property [39] and sustained release property. Results show that as the proportion of* Azadirachta indica* fruit mucilage increases, the overall time of release of the drug from the matrix tablet also increases [40].

### 2.12. Aloe Mucilage

Aloe mucilage is obtained from the leaves of* Aloe barbadensis* (family:* Liliaceae*). It contains arabinan, arabinorhamnogalactan, galactan, galactogalacturan, glucogalactomannan, galactoglucoarabinomannan, and glucuronic acid containing polysaccharides [41]. A controlled delivery system of glibenclamide using aloe mucilage was studied. Various formulations of glibenclamide with* Aloe barbadensis* Miller leaves mucilage were prepared by direct compression technique. The formulated matrix tablets were found to have better uniformity of weight and drug content with low statistical deviation. The swelling behaviour and* in vitro* release rate characteristics were studied. The dissolution study proved that the dried* Aloe barbadensis* Miller leaves mucilage can be used as a matrix forming material for making controlled release glibenclamide matrix tablets [42].

### 2.13. *Moringa oleifera* Gum

Gum is obtained from exudes of stem of* Moringa oleifera* (family:* Moringaceae*). The gum is a polyuronide constituting of arabinose, galactose, and glucuronic acid in the preparation of 10 : 7 : 2, rhamnose present in traces [43]. Studies were performed on this gum for its gelling property. The gelling concentration of the gum was found to lie between 7 and 8.5% w/v. The gels exhibited pseudoplastic flow and viscosity were found to be ideal for topical application [43], binding property [44], and release retardant property. Different batches of tablet were prepared and evaluated for drug release. It was observed that drug release increased with increasing proportions of the excipient and decreased proportion of the gum. Release mechanism was found to be Fickian [44]. Gum was also studied for its disintegrating property. Different batches of tablets were formulated varying them by quantity of the gum. It was observed that wetting time decreased with the increase in concentration of gum in formulation; thus disintegration time of tablet formulation prepared from gum was found lesser as compared to tablet formulation prepared from synthetic disintegrant like starch, sodium glycolate (SSG), and croscarmellose sodium (CCS) [45].

### 2.14. Gum Damar

Gum damar is a whitish to yellowish natural gum produced by tapping trees of* Shorea wiesneri* (Family:* Dipterocarpaceae*). It contains about 40% alpha-resin (resin that dissolves in alcohol), 22% beta-resin, 23% dammarol acid, and 2.5% water. Studies were performed on gum damar for its sustained release matrix forming property. Drug release from the matrix showed sustained drug delivery beyond 10 hour. [46]. Microencapsulating property of the gum was also evaluated. The increase in gum : drug ratio showed an increase in particle size, encapsulation efficiency and decrease in drug release rate [47]. It has been used also for water-resistant coating and in pharmaceutical and dental industries for its strong binding properties.

### 2.15. Gum Copal

Gum copal is a natural resinous material of plant* Bursera bipinnata* (family:* Burseraceae*). Copal resin contains agathic acid along with ciscommunic acid, transcommunic acid, polycommunic acid, sandaracopimaric acid, agathalic acid, monomethyl ester of agathalic acid, agatholic acid, and acetoxy agatholic acid [48]. Copal gum has been evaluated as matrix-forming material for sustaining the drug delivery. In an independent study copal resin was used as a film forming agent. Films showed good swelling property. It was concluded that it can be used as a coating material for sustained release and colon targeted drug delivery. Film was prepared using gum copal and its swelling studies were performed in different phosphate buffer (pH 4.5, pH 6.0, and pH 7.4); significant swelling was found in pH 7.4 so colon can be targeted [49].

### 2.16. Moi Gum

Moi gum is obtained from leaves, stems, fruits, and bark of the stem* Lannea coromandelica* (family:* Anacardiaceae*). This gum is yellowish white colour in fresh and on drying becomes dark. Gum ducts are present in leaves, stems, and fruits and are most abundant in the bark of the stem [50]. The roots contain cluytyl ferulate; heartwood gives lanosterol; bark, dlepi-catechin, and (+)-leucocyanidin; flowers and leaves, ellagic acid, quercetin, and quercetin-3 arabinoside. Flowers also contain isoquercetin and morin. Leaves in addition contain beta-sitosterol, leucocyanidin, and leucodelphinidin. Moi gum was evaluated as microencapsulating agent and release rate controlling material. Microspheres were prepared by solvent evaporation technique. Moi gum produced microspheres having acceptable size and morphology. Microspheres formulated using moi gum showed sustained release beyond 10 hours in comparison to guar gum but when used in 1 : 1 ratio microspheres showed more sustained release [51].

### 2.17. Kondagogu Gum

Kondagogu gum or hupu gum is a naturally occurring polysaccharide derived as an exudate from the tree* Cochlospermum religiosum* (family:* Bixaceae*). Gum contains rhamnose, galacturonic acid, glucuronic acid, b-D galactopyranose, a-D-glucose, b-D-glucose, galactose, arabinose, mannose, and fructose [52]. Studies were performed on kondagogu gum for its gastric floating property. The polymer concentration, concentration of sodium bicarbonate, and that of pharmatose to the weight of drug and polymer were selected as independent variables. Cumulative percent drug released at 12 hrs was selected as dependent variable. The release rate decreased as the proportion of hupu gum increased [53]. Hupu gum was also evaluated for its mucoadhesive microcapsule forming property. All microspheres showed good mucoadhesive property in* in vitro* wash of test.* In vitro* drug release studies showed that the guar gum had more potentiality to retard the drug release compared to other gums and concentrations. Drug release from the microspheres was found to be slow and following zero order release kinetics with non-Fickian release mechanism, stating that release is depended on the coat : core ratio and the method employed [54].

### 2.18. Phoenix Mucilage

Phoenix mucilage is obtained from the dried fruit of* Phoenix dactylifera* (family:* Palmaceae*). Carbohydrates make up to 44–88% of the fruit which include mainly reducing sugars such as fructose, sucrose, mannose, glucose, and maltose in addition to small amounts of polysaccharides such as pectin (0.5–3.9%), starch, and cellulose. Binding properties of date palm mucilage were successfully evaluated. The tablets manufactured using phoenix mucilage were found to be less friable than tablets manufactured using acacia and tragacanth. As the concentration of the gum increased the binding ability improved, producing good uniformity in weight and hardness of the tablets [55].

### 2.19. *Cassia tora* Mucilage

*Cassia tora* mucilage derived from the seeds of* Cassia tora* (family:* Caesalpiniaceae*). The primary chemical constituents of Cassia include cinnamaldehyde, gum, tannins, mannitol, coumarins, and essential oils (aldehydes, eugenol, and pinene); it also contains sugars, resins, and mucilage among other constituents [56]. Studies were performed on* Cassia tora* mucilage for its binding property. It was observed that increasing the concentration of mucilage increases hardness and decreases the disintegration time of the tablets which were formulated with different concentrations of* cassia tora* gum [57]. This mucilage was also evaluated for its suspending agent. The suspending ability of* Cassia tora* mucilage was compared with that of tragacanth, acacia, and gelatin. The suspending ability of all the materials was found to be in the order:* Cassia tora* > tragacanth gum > acacia gum. Gelatin results suggest that suspending action of the mucilage is due to high viscosity of the gum [58].

### 2.20. Bhara Gum

Bhara gum is a yellowish natural gum extracted from the bark of* Terminalia bellerica* (family:* Combretaceae*). Main chemical constituents are tannins which mainly include ß-sitosterol, gallic acid, ellagic acid, ethyl gallate, galloyl glucose, and chebulaginic acid. A new sustained release microencapsulated drug delivery system employing bhara gum has been proposed. The microcapsules were formulated by ionic gelation technique using famotidine as the model drug. The effect of different drug: bhara gum ratio drug release profile was examined and compared with guar gum. Microcapsules employing bhara gum exhibited slow release of famotidine over 10 hour [59].

### 2.21. Mimosa Mucilage

Gum is obtained from seeds of* Mimosa pudica* (family:* Mimosaceae*). Seed mucilage is composed of D-xylose and D-glucuronic acid. Mimosa seed mucilage hydrates and swells rapidly on coming in contact with water. A controlled delivery system for diclofenac sodium using Mimosa seed mucilage was studied. In this study different batches of tablets were formulated and their drug releases were checked. It was observed that as the proportion of the* Mimosa pudica* seed mucilage increases, there is a decrease in release of drug, the mechanism of release being diffusion for tablets containing higher proportion of mucilage, and a combination of matrix erosion and diffusion for tablets containing smaller proportion of mucilage. Studies showed that as the proportion of the mucilage increased, there was a corresponding increase in increase in percent swelling and decrease in percent erosion of the tablets [60].

### 2.22. *Mimosa scabrella* Gum

Gum is obtained from seeds of* Mimosa scabrella* (family:* Mimosaceae*). Gum is highly hydrophilic galactomannan that provides 20–30% of galactomannan (G) with a mannose : galactose ratio of 1.1 : 1. Studies were performed on* Mimosa scabrella* gum for its controlled release matrix forming property. In this study it was observed that drug release decreased with the increase of polymer concentration and 25% w/w of gum showed excessive sustained release effect. The release mechanism was a combination of diffusion and relaxation [61].

### 2.23. Dendrophthoe Mucilage

Dendrophthoe mucilage is obtained from dried as well as fresh stem parasite of* Dendrophthoe falcate* (family:* Loranthaceae*) on* Magnifera indica *(family:* Anacardiaceae*). Mucilage of* Dendrophthoe falcata* was evaluated as a binder for pharmaceutical dosage forms wet granulation was employed to make tablets with* Dendrophthoe falcate* mucilage. Different concentrations of mucilage were used in formulation. It was observed that 6% w/w binder concentration showed more optimum results as tablet binder [62].

### 2.24. Cocculus Mucilage

Mucilage is obtained from leaves of* Cocculus hirsute* (family:* Menispermaceae*). Mucilage contains polysaccharides and a gelatinous type of material. Leaves are used topically as emollient and demulcent. It has been nontoxic to human skin [63]. Gelling property of this mucilage was studied. This was a comparative study. Flurbiprofen was used as a model drug for the formulation of gel. Marketed flurbiprofen gel and gel prepared from* Cocculus hirsute* leaf powder were compared and both the gels were evaluated for anti-inflammatory property. It was observed that the quantity of drug released from prepared test gel and its anti-inflammatory activity was found to be more than that of marketed gel [64].

### 2.25. Hakea Gum

Hakea gum is a dried exudate from the plant* Hakea gibbosa* (family:* Proteaceae*). Gum contains glucuronic acid, galactose, arabinose, mannose, xylose which is 12 : 43 : 32 : 5 : 8. The exuded gum is only partly soluble in water [65]. Gum was investigated as a sustained release and mucoadhesive component in buccal tablets. These results demonstrate that* Hakea gibbosa*, not only may be used to sustain the release but also can act as bioadhesive polymer. In this study, time required for 90% of the drug was used as basis for comparison. It was observed that formulation which did not contain hakea gum showed 90% release of the drug in about 14 minutes. While when hakea gum was used in concentration of 32 mg per tablet, it was seen that 90% release of the drug took place in around 165 minutes. Also when tablets were directly compressed using hakea gum, for 32 mg gum per tablet, 90% release took place in 405 minutes [66].

### 2.26. Grewia Gum

Grewia gum is a polysaccharide derived from the inner bark of the edible plant* Grewia mollis* (family:* Tiliaceae*). The gum consists of glucose and rhamnose as the main monosaccharide components and galacturonic acid as the main sugar acid [67]. Studies were performed on grewia gum for its binding property, compressional property. In this study it was found that formulations containing grewia gum exhibited higher degree of packing than those containing PVP. Grewia gum was also found to improve fluidity granules than PVP. [68]. Studies were also carried out on matrix forming property of this gum. In this study tablets containing different concentrations of grewia gum were compressed by direct compression technique and were evaluated.* In vitro* drug release studies reveal that grewia gum can control the release of cimetidine from tablets for up to 12 hours. There was synergy between grewia gum and HPMC in delaying the release of cimetidine from tablets [69] and film forming property [70].

### 2.27. Mango Gum

Mango gum is a dried gummy exudate polysaccharide obtained from the bark of* Mangifera indica* (family:* Anacardiaceae*). Studies were performed on mango gum for its binding [71], sustained release [72]. Disintegrating property of this gum was also studied. Tablets containing this gum showed good appearance and better drug release. The study further revealed a poor relation between the swelling index and disintegrating efficiency [73]. Mouth dissolving tablets were prepared using this gum [73].

### 2.28. Olibanum Gum

Olibanum gum is a dried, gummy exudation obtained from* Boswellia serrate* (family:* Burseraceae*). Gum olibanum is used as an anti-inflammatory remedy and recent studies have found positive influence of olibanum on rheumatism. Its composition and chemical characteristics depend on its three principal origins: Aden/Somalia, Eritrea, and India which contains approximately 5–9% oil content, 13–17% resin acids, 20–30% polysaccharides, 40–60% boswellic acid. Studies were performed on olibanum gum for its sustained release matrix forming [74], binding [75]. Olibanum resin coated microcapsules were formulated by emulsification solvent evaporation method. It was observed that drug release from the resin-coated microcapsules was slow over 24 hours and depended on core : coat ratio, wall thickness, and size of the microcapsules [76].

### 2.29. Terminalia Gum

Terminalia gum exudates are from the incised trunk of the tree* Terminalia randii* (family:* Combretaceae*). Extracts of the stem and bark of* Terminalia randii* are used in the treatment of dysentery, diarrhea, hemorrhoids, and wounds. Gum exudates obtained from* Terminalia randii* have been evaluated as binding agent. The results showed that the crushing strength and crushing strength friability ratio increased with increase in polymer concentration while friability decreased [77].

### 2.30. Cordia Mucilage

Cordia mucilage is obtained from raw fruits of* Cordia obliqua* (family:* Boraginaceae*). Cordia mucilage can be used as expectorant, is effective in treating lung disease and raw gum can be used in gonorrhoea. Studies were performed on cordia mucilage for binding and emulsifying properties [78].

### 2.31. Ocimum Mucilage

Ocimum mucilage is obtained from the seeds of* Ocimum americanum* commonly called* Ocimum canum* (family:* Lamiaceae*). Mucilage contains xylose, arabinose, rhamnose, and galacturonic acids [79]. The mucilage was found to have disintegrating property. The disintegration time for tablet formulations prepared using ocimum mucilage was less than tablets that were prepared by using starch as a disintegrant [80].

### 2.32. Konjac Glucomannan

Konjac glucomannan is extracted from the tubers of* Amorphophallus konjac* (family:* Araceae*). Konjac glucomannan contains D-glucose and D-mannose in the ratio 1 : 1.6 [81]. Studies were performed on konjac glucomannan for its gelling properties [82].

## 3. Conclusion

The use of natural gums for pharmaceutical applications is attractive because they are economical, readily available, nontoxic, capable of chemical modifications, potentially biodegradable, and with few exceptions, also biocompatible. Majority of investigations on natural polymers in drug delivery systems centre around polysaccharides. Natural gums can also be modified to have tailor-made products for drug delivery systems and thus can compete with the synthetic excipients available in the market. Though the use of traditional gums has continued, newer gums have been used, some of them with exceptional qualities. There is huge scope for research on newer gums and mucilages obtained from plants and could be further exploited in future as a novel natural polymer for development of different drug delivery systems in pharma industry.

## Figures and Tables

**Table 1:** Preliminary confirmatory tests for dried gums and mucilage.

Test	Observation	Inferences
*Molisch's test*: (100 mg dried mucilage powder + Molisch's reagent + conc. H_2_SO_4_ on the side of a test tube)	Violet green colour observed at the junction of the two layers	Carbohydrate present

*Ruthenium test*: Take a small quantity of dried mucilage powder, mount it on a slide with ruthenium red solution, and observe it under microscope.	Pink colour develops	Mucilage present

*Iodine test*: 100 mg dried mucilage powder + 1 mL 0.2 N iodine soln.	No colour observed in solution	Polysaccharides present (starch is absent)
